# Pennsylvania State Dental Society

**Published:** 1891-06

**Authors:** 


### PENNSYLVANIA STATE DENTAL SOCIETY.

(For Dr. Leffmann's paper, see page 383.)

*Dr. L. Ashley Faught.*—Mr. President and Gentlemen,—I have listened with the deepest interest to the reading of this admirable paper. The time of its presentation seems to me to be most opportune. The subject of dental education has earnestly engaged the attention of the profession at different periods during the last twenty years. And each new outlook seems to have been taken at the beginning of each decade.

I conceive that our highest good lies not in developing either the mental or the mechanical at the expense of the other, but in a wise and judicious blending of the two. While it is true that ours is a surgico-artistic profession, and that mechanical operation constitutes the largest part of our duties, that we are engaged more with the mechanical than with the medical, more with the prosthetic than with the therapeutic, yet the coming dentist will practise as an adviser as well as an operator, and must be a scientific man. What he should seek to do is to blend the science with the practical, but this practical education cannot be taught, it can only be acquired.

There are, however, concomitant evils connected with dental education which, from our stand-point outside the rank of teachers, demand indication.

We want and must have, at the present time, less rivalry between our institutions of learning. The effort should not be for popularity, or to rank as the best; but the aim should be the same in all,—to help one another preserve the honor of the degree, and to properly prepare material for the dental ranks. The list of matriculates will bear large pruning, for many are admitted who show a most unhealthy tone at the very start by inquiring "how to get a degree with the least expenditure of time and money."

I believe, too, that a more healthy tone could be imparted to dental colleges if their boards of trustees were composed of their own local alumni. Operating in somewhat similar manner, much can be expected from the work of those State boards of registration and examination in dentistry, which may be in the future created to exist under such State dental laws as the New Jersey Society succeeded in having passed in April of this year.

*Dr. Gerhart.*—In the final examinations, deficiencies in one department should not be condoned because the candidate for graduation manifests proficiency in other departments.

The matriculate should have not only a good English education, but should also have some knowledge of Latin and Greek, because upon these technical terms are based.

DISCUSSION OF DR. LIBBEY'S PAPER ON "IMMEDIATE ROOT-FILLING."

(For Dr. Libbey's paper, see page 388.)

Dr. Roberts desired to know what becomes of the chlora-percha forced through the apical foramen.

Dr. Libbey, replying, quoted Dr. Jennings, of Cleveland, Ohio, as authority for the statement that the chlora-percha becomes encysted. He does not think it is absorbed by the circulation.

Dr. Roberts asked further for information as to whether Dr. Libbey placed entire dependence for antisepticism upon the chlora-percha, or whether its injection is preceded by the application of a disinfectant.

Dr. Libbey stated that the paper explains that in its reference to the use of permanganate of potassium, alcohol, hot air, and wood creosote. He thought these were sufficiently disinfectant.

Dr. Ward said that in his experience the principal difficulty met with in the treatment of these cases exists in the fact that patients cannot always be relied upon to obey directions, and to keep appointments when roots are not filled immediately. They want immediate relief when a tooth aches, and when the pain is allayed they neglect attending to it afterwards unless pain recurs. He has found oxide of zinc valuable as an agent in the immediate filling of roots.

